# Monofloral Corn Poppy Bee-Collected Pollen—A Detailed Insight into Its Phytochemical Composition and Antioxidant Properties

**DOI:** 10.3390/antiox12071424

**Published:** 2023-07-14

**Authors:** Aleksandar Ž. Kostić, Danijel D. Milinčić, Bojana Špirović Trifunović, Nebojša Nedić, Uroš M. Gašić, Živoslav Lj. Tešić, Sladjana P. Stanojević, Mirjana B. Pešić

**Affiliations:** 1 Department of Chemistry and Biochemistry, Faculty of Agriculture, University of Belgrade, Nemanjina 6, 11080 Belgrade, Serbia; 2 Department for Pesticides and Herbology, Faculty of Agriculture, University of Belgrade, Nemanjina 6, 11080 Belgrade, Serbia; 3 Department for Breeding and Reproduction of Domestic and Bred Animals, Faculty of Agriculture, University of Belgrade, Nemanjina 6, 11080 Belgrade, Serbia; 4 Department of Plant Physiology, Institute for Biological Research Siniša Stanković-National Institute of Serbia, University of Belgrade, Bulevar Despota Stefana 142, 11060 Belgrade, Serbia; 5 Department of Analytical Chemistry, Faculty of Chemistry, University of Belgrade, Studentski Trg 12–16, 11000 Belgrade, Serbia; ztesic@chem.bg.ac.rs

**Keywords:** antioxidants, bee-collected pollen, phytochemical composition, phenolics, polyamides, alkaloids, fatty acids, *Papaver rhoeas* L.

## Abstract

The aim of this study was to compile a detailed phytochemical profile and assess the antioxidant properties of bee-collected pollen (PBP) obtained from corn poppy (*Papaver rhoeas* L.) plants. To achieve this, a lipid fraction was prepared for quantifying fatty acids using GC-FID. Extractable and alkaline-hydrolysable PBP fractions (obtained from a defatted sample) were used to determine the qualitative and quantitative profiles of phenolic compounds, phenylamides and alkaloids using UHPLC/Q-ToF-MS. Additionally, various spectrophotometric assays (TAC, FRP, CUPRAC, DPPH^⦁^) were conducted to evaluate the antioxidant properties. Phenolic compounds were more present in the extractable fraction than in the alkaline-hydrolysable fraction. Luteolin was the predominant compound in the extractable fraction, followed by tricetin and various derivatives of kaempferol. This study presents one of the first reports on the quantification of tricetin aglycone outside the Myrtaceae plant family. The alkaline-hydrolysable fraction exhibited a different phenolic profile, with a significantly lower amount of phenolics. Kaempferol/derivatives, specific compounds like ferulic and 5-carboxyvanillic acids, and (epi)catechin 3-*O*-gallate were the predominant compounds in this fraction. Regarding phenylamides, the extractable fraction demonstrated a diverse range of these bioactive compounds, with a notable abundance of different spermine derivatives. In contrast, the hydrolysable fraction contained six spermine derivatives and one spermidine derivative. The examined fractions also revealed the presence of seventeen different alkaloids, belonging to the benzylisoquinoline, berberine and isoquinoline classes. The fatty-acid profile confirmed the prevalence of unsaturated fatty acids. Furthermore, both fractions exhibited significant antioxidant activity, with the extractable fraction showing particularly high activity. Among the assays conducted, the CUPRAC assay highlighted the exceptional ability of PBP’s bioactive compounds to reduce cupric ions.

## 1. Introduction

Nutrition is one of the most important aspects of our lives, but it is also one of the most delicate. Modern consumers are increasingly seeking high-quality food from natural sources. Consequently, food production has become a rapidly growing and demanding sector of the industry. Meeting market demand requires not only increased food production, but also improved quality. Therefore, functional food has gained popularity in modern food science, as it refers to novel food products with enhanced benefits in our diet. Unlike the commonly used definition, the latest definition of functional food has expanded its scope to include the following: “Functional food is a novel food that has been formulated to contain substances or live microorganisms that have the potential to enhance health or prevent diseases, at a concentration that is both safe and sufficiently high to achieve the intended benefit” [1]. Bee-collected pollen is an invaluable source of essential nutrients and bioactive compounds for both bees and humans, earning it the reputation of being a “treasure trove” of nature [2]. The application of bee-collected pollen can significantly improve the functionality of food, benefiting both biological/health and techno-functional properties [3,4]. Among the various bioactive constituents of bee-collected pollen, phenolic compounds play a crucial role, and they have been extensively studied in the last decade [5,6,7,8,9,10]. Apart from phenolics, different polyamines are extremely important plant secondary metabolites with expressed bioactivity. These compounds are important for combating plant stress, enabling the plant to increase abiotic stress tolerance [11,12]. They are also important in human health since some of them, like spermidine, can express strong activity against age-related diseases [13]. Spermidine has also shown promise as a potential candidate for reducing the risk of cancer in humans [14]. Pollen, due to its sensitivity, serves as an excellent source of polyamines and their derivatives, formed predominantly with phenolic acids, known as phenylamides. These derivatives in pollen also express an important bioactivity. For instance, phenylamides from *Quercus mongolica* bee-collected pollen have demonstrated strong anti-tyrosinase activity [15]. Furthermore, polyamides of hydroxycinnamic acids have been found to protect sunflower pollen from fungal activity [16]. Despite being overlooked in pollen research for years, polyamines and their phenyl derivatives have recently gained significant interest during the last couple of years, as evidenced by several excellent research articles [17,18,19,20]. However, there is still a lack of data on monofloral bee-collected pollen (pollen consisting of more than 80% of a single pollen type), which can provide samples with consistent chemical composition. During foraging, bees selectively visit plants based on factors such as availability (depending on the season and geographical area), nectar richness, pollen quantity and protein content [21,22]. With this in mind, the aim of this study was to evaluate the phytochemical composition and antioxidant activity of monofloral bee-collected pollen obtained from corn poppy (*Papaver rhoeas* L., Papaveraceae) plants collected in Serbia. Currently, there is lack of data available on corn poppy bee-collected pollen. Only one report from Slovakia reporting about similar pollen originating from poppy (*Papaver somniferum* L.) confirmed its significant antioxidant activity expressed through DPPH radical scavenging (75.9% of inhibition), total antioxidant capacity (TAC) determined via an in vitro phosphomolybdenum assay (3.5 mg/mL) and with the total phenolic content at 817.3 mg/kg [23]. Additionally, Zhou et al. [24] identified three different flavonoid glycosides in different ranges with quercetin-3-*O*-β-D-glucosyl-(2→l)-β-glucoside as a possible chemical marker for poppy bee-collected pollen. Also, the most recent article revealed biofunctional properties of proteins and peptides obtained from Persian poppy pollen (*Papaver bracteatum*) [25]. To further characterize corn poppy bee-collected pollen (PBP), the present study aimed to determine the following parameters: (1) fatty acid composition of the lipid fraction; (2) general phytochemical composition (total carotenoid, phenolic, flavonoid, dyhydroxicinammic acid derivative content); (3) phenolic (qualitative and quantitative), phenylamide (qualitative) and alkaloid (qualitative) profiles; and (4) antioxidant activity expressed through TAC, DPPH radical scavenging capability, Ferric-Reducing Power (FRP) and Cupric-Reducing Antioxidant Capacity (CUPRAC) assays.

## 2. Materials and Methods

### 2.1. Collection and Extraction Procedures

**Figure 1:**
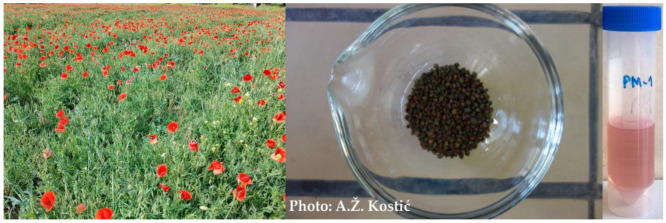
Appearance of corn poppy plants, obtained corn poppy bee-collected pollen and extractable fraction.

Corn poppy bee-collected pollen (PBP) was obtained from the private apiary of professor N. Nedić, located near Belgrade (the capital of Serbia), in May 2021. In order to obtain a pure and botanically homogenous sample, pollen traps were introduced at the hive’s entrance. Pollen from the traps was separated, checked for purity and collected every day. There were no other pastures nearby. The obtained sample was uniform in terms of color and pollen-grain shapes, confirming its monofloral origin and composition (Figure 1).

**Figure 2:**
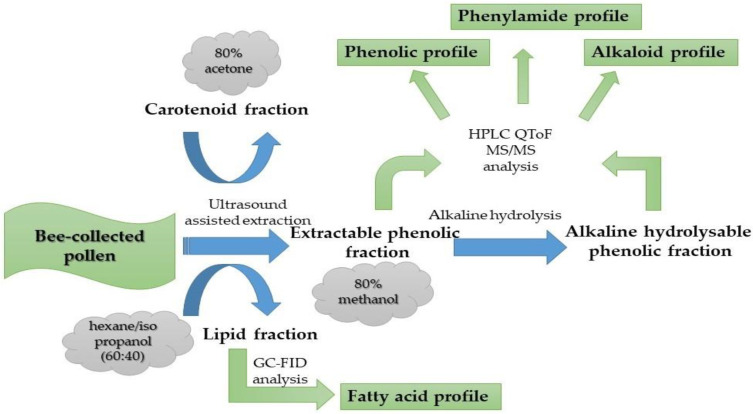
Illustration of experimental extraction procedure followed by applied analytical techniques.

The PBP extraction procedure (Figure 2) followed the method detailed in our previous research [26] with one modification—the ultrasound-assisted extraction pre-treatment was extended to 1 h instead of 30 min. Prolonged ultrasound-assisted extraction should have a positive effect on the improved extraction of bioactive components via the additional destruction of pollen membranes—exine and intine. As a result, four different extracts were obtained and used: (1) lipid PBP extract was used for the determination of fatty-acid composition; (2) extractable PBP phenolic fraction was used to determine the composition of phenolic compounds, phenylamides and alkaloids; (3) alkaline-hydrolysable PBP extract (referred to as bound PBP fraction) was applied to determine phenolic, phenylamide, and alkaloid composition; and (4) PBP extract in 80% acetone was used to determine the total carotenoid content.

### 2.2. General Phytochemical Characterization

Corn poppy bee-collected pollen (PBP) extracts obtained for extractable and bound phenolic fractions were examined and characterized as previously described [26] to determine the following general phytochemical parameters: (1) total phenolic content (TPC); (2) total flavonoid content (TFC); (3) total hydroxycinnamic-acid-derivative content (HCA); and (4) total carotenoid content (TCC). All results are expressed as mg of adequate equivalents per g of dry weight (dw) sample except for TCC (µg/g dw) [26]. 

### 2.3. A Detailed Profiling of Obtained Extracts

Corn poppy bee-collected pollen (PBP) extracts obtained for lipid, extractable and bound phenolic fractions were studied to determine detailed PBP profiles. For fatty-acid composition determination, the previously described method was applied [27]. The obtained results are expressed as % of total fatty acids determined in the sample.

The following procedure was applied for the detailed profiling of phenolics, phenylamides and alkaloids present in PBP. The analyses were carried out using an Agilent 1290 Infinity ultra-high-performance liquid chromatography (UHPLC) system coupled with quadrupole time-of-flight mass spectrometry (6530C Q-ToF-MS) from Agilent Technologies, Inc., CA, USA. The chromatographic separation was conducted at 40 °C on a Zorbax C18 column (2.1 × 50 mm, 1.8 µm) from Agilent Technologies, Inc., CA, USA. The mobile phase consisted of a mixture of (A) ultrapure water and (B) acetonitrile (MS grade), both A and B containing 0.1% HCOOH (MS grade). The flow rate was kept constant at 0.3 mL/min, and the injection volume was 5 µL. The gradient elution program began with 2% solvent B for the first 2 min, which then reached 98% B over the next 17 min, and over the next 5 min the gradient returned to its initial state (2% B) to re-equilibrate the column. The QToF-MS system was equipped with a Dual Agilent Jet Stream electrospray ionization (ESI) source operating in both positive (ESI^+^) and negative (ESI^−^) ionization modes. The operation parameters for ESI were set as follows: nebulizer pressure of 45 psi, a drying gas temperature of 225 °C and a flow rate of 8 L/min, sheath gas temperature of 300 °C and sheath gas flow 10L/min, capillary voltage of 2500 V, fragmentor energy of 175 V, skimmer voltage of 65 V, octopole RF Peak at 750 V. The QToF-MS system recorded the spectra over the *m*/*z* range 50–1700, with a scan rate of 2 Hz. Data-dependent acquisition (DDA) was employed for suspect screening using the Auto MS/MS acquisition mode with collision energy at 30 eV. Parameters for the Auto MS/MS mode were as follows: *m*/*z* = 50–1700, scan rate 1 spectra/sec. Agilent MassHunter software was used for data evaluation and analysis. Phenolics were identified based on their monoisotopic mass and MS fragmentation, while they were quantified via direct comparison with available standards. Due to the lack of some specific standards, the quantities of the individual phenolic derivatives were quantified using available standards and expressed as μg/kg of the sample DW. Table S1 shows a list of phenolic compounds used for quantification, together with their equation parameters and correlation coefficient (r^2^). Anthocyanin, phenylamide derivatives and alkaloids were identified based on their monoisotopic mass and MS^2^ fragmentation and confirmed using previously reported data found in the literature. Accurate masses of components were calculated using ChemDraw software (version 12.0, CambridgeSoft, Cambridge, MA, USA). All the results for the content of phenolic compounds are given as µg/kg dw of the sample. Equations used for quantification of identified phenolic compounds are given in the supplementary file (Table S1).

### 2.4. Antioxidant Properties of PBP

Corn poppy bee-collected pollen extracts, containing extractable and bound fractions of bioactive compounds, were subjected to the examination of antioxidant properties by applying four different assays: in vitro phosphomollybdenum total antioxidant capacity (TAC), DPPH (α, α-diphenyl-β-picrylhydrazyl) radical scavenging activity, Cupric-Reducing Antioxidant Capacity (CUPRAC) and Ferric-Reducing Power (FRP), following the method detailed in our previous research [26].

### 2.5. Statistical Analysis

Results for general phytochemical characterization, antioxidant properties and fatty-acid composition were performed in triplicate and presented as means ± standard deviation (SD). For evaluation of statistical differences between the means, we applied *t*-tests (*p* < 0.05) (Statistica software version 12.0, StatSoft Co., Tulsa, OK, USA).

## 3. Results

### 3.1. General Phytochemical Composition

Based on different spectrophotometric methods, it is possible to determine the general and outline content for different groups of phytochemicals. In this case, the results obtained for the PBP content of the determined phytochemicals are presented in Table 1.

In all cases, phenolic compounds were predominantly present in the extractable fraction compared with the bound fraction. The extractable fraction exhibited the highest content of total phenolics (11.6 mg/g GAE dw) and flavonoids (12.8 mg/g QE dw). On the other hand, hydroxycinnamic acid derivatives were the least represented subclass of phenolics in both fractions, ranging from 1.14 to 5.96 mg/g CGAE dw. Interestingly, the spectrophotometric assay failed to quantify the total flavonoids in the bound fraction. In addition, the total carotenoid content determined for corn poppy PBP was 65.05 µg/g dw. 

### 3.2. UHPLC Phenolic Profile of PBP Extracts

The obtained results derived from UHPLC-QToF-MS analysis are presented in Table 2.

According to the obtained results, it can be observed that phenolics belonging to four subclasses were identified and quantified: phenolic acids and derivatives, flavones, flavanones and derivatives and flavonols and derivatives. In total, fifty metabolites (forty-eight phenolics and two organic acids) were identified and quantified: thirteen phenolic acids/derivatives, five flavones, two flavanones, one ester of (epi)catechin and four anthocyanin derivatives. However, among phenolics, flavones, flavonols and derivatives were strongly predominant, with a remarkable diversity of twenty-three different compounds. Interestingly, (epi)catechin 3-O-gallate was identified and quantified only in the bound fraction (50.81 mg/kg dw), as well as two simple organic acids: citric (in both fractions) and gluconic (in the extractable fraction). The highest content in the extractable fraction was observed for two flavones, luteolin (4398.1 mg/kg dw) and tricetin (3048.97 mg/kg dw), followed by kaempferol (414.85 mg/kg dw) and derivatives: kaempferol 3-O-(2″-pentosyl)hexoside (885.46 mg/kg dw) and kaempferol 3-O-(6″-pentosyl)hexoside (714.93 mg/kg dw). Furthermore, two quercetin derivatives (487.11–577.59 mg/kg dw) were quantified in significant quantities, as well as isorhamnetin as an aglycone (321.30 mg/kg dw). Once again, this confirmed the great predominance of flavones and flavonols and, in particular, their glycosides as phenolic compounds present in bee-collected pollen. Unlike the extractable fractions, the bound fractions contained a significantly lower amount of different phenolics with much lower diversity. Among them all, kaempferol 3-O-(2″-pentosyl)hexoside (700.17 mg/kg dw) and its aglycone (364.72 mg/kg dw) were predominant. Moreover, some phenolic acids were present only in the bound fraction, like ferulic acid (125.32 mg/kg dw) and 5-carboxyvanillic acid (610.81 mg/kg dw), which were probably liberated from the cell-wall component due to the breaking of the chemical bonds caused by strong alkaline conditions [28,29,30]. Curiously, none of the quercetin and most of the isorhamnetin derivatives were present in the bound fraction. Due to the lack of appropriate standards, anthocyanin derivatives were only identified (Table 3).

It is worth noting that different anthocyanin derivatives were identified in the fractions. While the extractable fraction contained cyanidin 3-*O*-(6″-pentosyl)hexoside isomer I and delphinidin 3-*O*-(6″-*O*-hexosyl)hexoside, the bound fraction was characterized by the presence of cyanidin 3-*O*-(6″-pentosyl)hexoside isomer II and cyanidin 3-*O*-glucoside. These compounds (most probably delphinidin derivative) are likely responsible for the purple color of the extractable fraction prepared for analysis (Figure 1).

### 3.3. UHPLC Phenylamide (Derivatives) Profile of PBP Extracts

The obtained results derived from UHPLC-QtoF-MS analysis are presented in Table 4.

In total, twenty-seven phenylamide derivatives were identified, while two were partially identified as coumaroyl derivatives. Among them, phenyl derivatives of spermine were predominant (eighteen compounds), followed by five phenyl derivatives of putrescine and four compounds originating from spermidine. Based on the phenolic moiety present in these phenylamides, a significant predominance was observed for different coumaroyl derivatives, followed by an acetyl, caffeoyl, feruloyl and benzoyl structural unit or in combination with them.

### 3.4. UHPLC Alkaloid Profile of PBP Extracts

The obtained results derived from UHPLC-QToF-MS analysis are presented in Table 5.

In total, seventeen different alkaloids were identified in both fractions belonging to three distinct alkaloid subclasses: benzylisoquinoline, berberine and isoquinoline types. All compounds were detected in the extractable fraction, whereas the bound fraction contained twelve different alkaloids.

### 3.5. Fatty-Acid Profile of PBP Extract

The obtained results derived from GC-FID analysis are presented in Table 6.

In total, nine different fatty acids (FAs) were identified and quantified. Based on the obtained results, it can be observed that the most abundant FA in lipid PBP extract was docosahexaenoic (DHA) acid (25.26%), followed by α-linolenic (22.98%) and stearic (13.72%) acids. A significant predominance of unsaturated fatty acids was observed, since almost 75% of all the FAs were different UFAs.

### 3.6. Antioxidant Properties of PBP Phenolic Extract

The obtained results for applied assays are presented in Table 7.

As can be seen from the given results, the extractable fraction exhibited significantly higher antioxidant activity in all applied assays. Both fractions showed a high ability to reduce Cu^2+^ ions, as determined by the CUPRAC assay. However, the bound fraction had a low total antioxidant capacity (0.92 mg/g AAE dw) as well as a low ability to reduce ferric ions (0.35 mg/g AAE dw).

## 4. Discussion

### 4.1. General Phytochemical Composition

Based on the results obtained for carotenoid content, it can be concluded, in line with the literature, that PBP possessed a significantly higher content of carotenoids compared with a monofloral coconut sample from Brazil (2.17–3.55 µg/g dw) [31] or an artichoke bee-collected pollen sample from Serbia (5.00 µg/g dw) [26]. This difference may be related to the lighter color intensity observed in both coconut and artichoke bee-collected pollen samples [26,31] compared with that in PBP. Among the different phenolic subclasses, spectrophotometric analyses showed a significantly higher content for all phenolics in the extractable fraction compared with the bound fraction. The obtained TFC (12.82 mg/g QE dw) in the extractable fraction was high, while in the bound fraction, total flavonoids were below the limit of detection. The obtained value was significantly higher or in line with previously published data on pollen samples from Brazil and Serbia, where the results for the extractable fractions ranged from 0.3 to 11 mg/g QE [10,26,32]. The only sample with a higher TFC value was the monofloral *Myrcia* bee-collected sample (17.5 mg/g QE) from the Brazilian state Rio Grande de Sul [32]. According to TPC results, the PBP extractable fraction contained a significantly higher amount of total phenolics compared with a sample with similar botanical origin—*P. somniferum* bee-collected pollen from Slovakia (0.82 mg/g GAE) [23]. However, the TPC result was in line with several bee-collected pollen samples from Brazil [32], particularly with monofloral *Mimosa caesalpiniifolia* bee-collected pollen (12.1 mg/g GAE dw), while it was significantly higher compared with that of coconut (~2 mg/g GAE dw) [31], sunflower (extractable—2.9–3.8 GAE dw) [10] and artichoke (extractable—5.3 mg/kg GAE dw; bound—0.5 mg/g GAE dw) [26] monofloral bee-collected pollen samples from Brazil and Serbia, respectively. Moreover, a bee-collected pollen sample from Morocco (*Coriandum sativum* + Cistaceae) showed a similar TPC value—13.73 mg/g GAE [33]. Unlike TPC and TFC assays, results for the HCA content in bee-collected pollen samples are quite rare. It was reported that artichoke bee-collected pollen contained 1.06 mg/g CGAE dw in the extractable fraction, while these derivatives were not observed in the bound fraction [26]. In both cases, the results were significantly lower compared with PBP. The presented comparative literature analysis once again confirms the importance of both the botanical and geographical origin of pollen samples for their content of different bioactive compounds.

### 4.2. UHPLC Phenolic Profile of PBP

There is a lack of data about the phenolic profiles of both poppy (*P. somniferum*) and corn poppy bee-collected pollen. To the best of our knowledge, the only available data are provided by Kačaniova et al. [34] for the *P. somniferum* sample collected in Slovakia. The authors reported the presence of four different aglycone flavonoids (luteolin, kaempferol, apigenin, quercetin) according to HPLC analysis, with a great prevalence of luteolin (1390.67 mg/kg dw). This is consistent with the data from the present research, as luteolin and other identified/quantified flavonoids were present in the PBP sample in the form of aglycone or some glycoside derivatives, with luteolin found in a concentration four times higher than the most prevalent compound. However, what is interesting is the presence of tricetin, another flavone found as the second most prevalent phenolic compound in the extractable fraction. Until know, this compound has been recognized as a taxonomic marker for Myrtaceae pollen samples [35] as well as for honey samples originating from the same botanical family [36]. However, a recent review article clearly stated that there is a strong possibility that this aglycone is occasionally overlooked in different samples due to the lack of modern and more precise techniques [36] such as QToF. In line with this and the results of the present study, recent research has provided detailed data on the phenolic profile of the *Perilla frutescens* L. (Lamiaceae) plant, confirming the presence of tricetin as an aglycone [37]. Additionally, it should be mentioned that there is a strong possibility that this aglycone is actually a precursor for the biosynthesis of alkaloids in *Papaver* plants [38]. Moreover, detailed phenolic profiles of different pollen samples originating from different areas and plants have revealed an astonishing diversity of different phenolic compounds/derivatives [19,32,39,40]. What is common in all the research studies is the predominant representation of flavonols, flavones and derivatives. Some of the compounds found in the current research have already been identified in different samples. For instance, *p*-coumaric (0.20 mg/g) and caffeic (0.15 mg/g) acids were determined in monofloral Moroccan bee-collected pollen samples originating from Apiaceae and Fabaceae plants, respectively [41]. Furthermore, similar to the current research, the authors found different quercetin and kaempferol glycosides (ranging from 1.19 to 1.77 mg/g) as predominant compounds [41]. In a polyfloral sample consisting mostly of *Cytisus stratius* and *Eucalyptus* sp. pollen from north-east Portugal, quercetin-3-*O*-rhamnoside and luteolin were identified as predominant phenolics [19]. Similarly, in bee-collected pollen samples from Brazil, isorhamnetin-3-*O*-(2″-*O*-rhamnosyl)glucoside, the same compound found in the PBP sample, was identified as one of phenolics in monofloral *Cocos nucifera* L. bee-collected samples [40]. Additionally, quercetin-3-*O*-arabinoside, identified in seven floral pollen samples originating from São Paolo, a Brazilian state [39], could respond to quercetin-3-*O*-pentoside, identified and quantified in the current research. In the same study, the presence of kaempferol-3-*O*-glucoside was also confirmed in all examined samples [39]. Interestingly, the most recent publication provided data on monofloral *Castanea* bee-collected pollen from the Iberian Peninsula (Galicia and northern Portugal) containing exclusively isorhamnetin derivatives (five compounds) as well as naringenin among the phenolics [42]. However, significant differences in other specific compounds found in PBP compared with the literature data can be attributed to the botanical origin of the samples. When comparing the extractable and bound phenolic fractions, it is clear that most phenolics in PBP are present in the extractable fractions, as a significant number of compounds were absent in the bound fraction or quantified in decreased amounts. Similarly, a lower amount of bound phenolics was observed in artichoke monofloral bee-collected pollen [26]. However, some of the compounds, particularly certain phenolic acids and (epi)catechin-3-*O*-gallate, were identified and quantified only in the bound fraction. One possible reason for this is their release from complex biomacromolecules such as sugars, proteins and polymers present in cell walls under strong alkaline conditions. This phenomenon is strongly related to the plant’s origin. For instance, it is well-known that in the case of cereals and wheat, the bound phenolic fraction can be predominant, accounting for up to 99% in cereal brans [29]. Regarding the identified anthocyanins in PBP, there are no available data on the presence of these phenolic subgroups in corn poppy pollen. In fact, there is a significant shortage of data on specific anthocyanins in pollen in general. Nevertheless, two independent studies have reported that anthocyanins are predominant compounds in the overall phytochemical composition (53.44–77.37 mg/L cyanidin-3-glucoside equivalents) of *Castanea*, *Cistus* and *Rubus* bee-collected pollen from Tuscany, Italy [43,44].

### 4.3. UHPLC Phenylamide Profile of PBP

This group of phenyl derivatives began to be examined in the last decade in bee-collected pollen samples, and the data have been published in several reports [15,19,39,40,41,45]. Similar to this current work, Zhang et al. [45] found a significant predominance of coumaroyl derivatives (thirty compounds) of spermine, spermidine and putrescine in monofloral samples from China, followed by six caffeoyl and four feruloyl derivatives. It was found that apricot monofloral bee-collected pollen contained predominantly different coumaroyl phenylamides, as well as sunflower pollen (but in a lower quantity), while rose bee-collected pollen was also characterized by a significant abundance of caffeoyl derivatives. On the other hand, the camellia sample contained a higher amount of feruloyl derivatives compared with the other samples. Bee-collected pollen obtained from *Quercus mongolica* was characterized by four different, specific, polyamine derivatives—mangolicine A, and mangolidine A, B and C [15]. Corresponding to the present study, different isomers of tricoumaroyl spermidine were identified in *Castanea* bee-collected pollen samples [42], although a prevalence of caffeoyl spermidine derivatives was observed, unlike PBP. Coconut bee-collected pollen from Brazil [40] also contained tricoumaroyl spermidine, as well as six additional spermidine derivatives different from PBP. What was specific for PBP was the significant presence of spermine derivatives as opposed to previous research studies, as well as the presence of an acetyl moiety in these structures. Related to this, tetracoumaroyl spermine (isomer) was identified and quantified (3.34 mg/g) as the most predominant bioactive compound in the bee-collected pollen sample, containing, with great predominance, grains of two plant species/genera—*Crepis capillaris* (Smooth hawksbeard) and *Plantago* sp. [19]. These isomers were also identified and quantified in polyfloral Cystaceae and Asteraceae samples from Morocco [41]. Clearly, a great diversity of these compounds can be linked to botanical origin. With some additional statistical analyses of all known data, there is a possibility that some of them can be used as chemotaxonomic markers. In the case of PBP, acetyl derivatives should be of particular interest since there is only one report about their presence in *Q. mongolica* bee-collected pollen [15]. In the case of PBP, a great diversity has been observed: acetyl spermine, five coumaroyl acetyl spermine isomers, caffeoyl acetyl spermine, three coumaroyl diacetyl spermine isomers, dicoumaroyl acetyl spermine, coumaroyl feruloyl acetyl spermine, dicoumaroyl diacetyl spermine, tricoumaroyl acetyl spermine and dicoumaroyl acetyl feruloyl spermine were identified. Interestingly, there is a significant absence of phenylamides in the bound fraction, as only seven spermine derivatives were identified, while all putrescine and spermidine derivatives were deficient, probably due to the strong alkaline conditions applied for compound liberations from chemical bonds. It was reported that, depending on the amide structure, alkaline conditions can enhance amide bond hydrolysis [46].

### 4.4. UHPLC Alkaloid Profile of PBP

In the case of PBP extracts, the presence of alkaloids is strictly related to their botanical origin and corn poppy’s chemical characteristics as alkaloid-rich plants. Specifically, different benzylizoquinoline alkaloids are present in corn poppy, with papaverine being the most important one [47]. In addition to papaverine, papaveraldine, a carbonyl derivative of papaverine, as well as papaverrubine E were also identified as specific corn poppy alkaloids in this study. Among pollen samples, the presence of alkaloids is not expected, except in the case of pyrrolozidine alkaloids [4,48]. They are predominantly specific for the pollen of *Echium*, *Senecio* and *Eupatorium* plants [48], representing undesirable components of pollen due to their toxicity. However, unlike these, other alkaloids also possess health-promoting properties such as antiviral activities [49]. In particular, the alkaloids detected in PBP extracts can actually exert beneficial effects for humans through consumption. For instance, isoquinoline alkaloids possess anticancer properties [50], while papaverine, per se, has been recognized as a miracle compound with different health benefits [51]. It has been demonstrated that local application of papaverine on patients with end-stage renal disease can induce a reduction in arteriovenous fistula maturation without any additional difficulties [52]. This literature evidence indicates that the presence of alkaloids in PBP does not have to be harmful in itself in the event of eventual consumption as a functional food ingredient. Of course, in this case, the question of dosage is quite important.

### 4.5. GC-FID Fatty-Acid Profile of PBP

Fatty acids are one of the most important nutrients present in bee-collected pollen, with great diversity observed depending on the botanical origin of the samples [31,53,54,55]. However, to the best of our knowledge, there are no data for the fatty-acid profile of corn poppy pollen, although there are extensive data on the lipid composition of poppy seeds and some other corn poppy plant parts. Based on the obtained results, it can observed that the lipid fraction of PBP is in line with the available data for poppy seeds, showing significant predominance of unsaturated FAs [56,57,58,59]. Nevertheless, the distribution of single acids differs in bee-collected pollen compared with poppy-seed composition. For instance, linoleic acid was predominantly found in seed samples [56,57,58], whereas in the case of PBP, docosahexaenoic acid (DHA) followed by α-linolenic acid were the main FAs. It has also been documented that α-linolenic acid is one of the predominant FAs in the leaves of corn poppy plants [60]. The significant presence of ω-3 FAs makes them an excellent source of these acids, which are important for balanced nutrition intake, along with ω-6 FAs, in the human diet. They are recommended to prevent the development of obesity [61]. Additionally, the significant share of DHA is extremely important since this FA has been recognized as one of the most important ω-3 FAs that can benefit both children and adults. It is documented to be important for brain development, the prevention of premature birth, cardiovascular diseases, as well as the improvement of cognitive function and vision in older people [62].

### 4.6. Antioxidant Properties of PBP Phenolic Extracts

The antioxidant properties of some pollen samples are mostly related to the content of different bioactive compounds, particularly phenolics. However, the importance of polyamines and their phenyl derivatives, which are identified in significant amounts in PBP extracts, should not be overlooked. It is well-known that polyamines can play a crucial role in a plant’s fight against oxidative stress by activating several antioxidant enzymes [12]. Therefore, expanded research should focus more attention on phenylamides as potential antioxidants. The examined PBP-extractable fraction exhibited significant antioxidant properties based on the results of all applied assays. According to the TAC value for the extractable fraction, it showed significantly higher total antioxidant capacity (28.92 mg/g AAE) compared with several samples from Morocco, where TAC values ranged from 3.98 to 9.03 mg/g AAE [33]. Possible reasons for the observed differences include different geographical and botanical origins (the Moroccan samples were all polyfloral) as well as a different extraction solvent (ethanol). The results of the FRP assay for the extractable PBP fraction were consistent with the results for a polyfloral sample from Portugal (5.0 mg/g GAE) predominantly originating from *C. striatus* and *Plantago* sp. [19]. However, the PBP-extractable fraction exhibited strong antioxidant activity in the CUPRAC assay (69.00 mg/g AAE), comparable to similar results for a polyfloral bee-collected pollen sample from Turkey—85.59 mg/g TE [63]. The bound PBP fraction also exhibited CUPRAC activity (22.78 mg/g GAE), unlike all other assays. Moreover, for several commercial pollen samples from Turkey, the authors reported significant CUPRAC results with the extractable fraction (6.25–64.88 µmol/g TE), lower than those with the hydrolysable fraction (69.16–192.96 µmol/g TE) [64]. It should be noted that the authors prepared the hydrolysable fraction differently, without strong alkaline conditions but with acidic hydrolysis. It is well-known that these conditions are favorable for CUPRAC assays [65]. Furthermore, it should be pointed out that significantly higher results for the CUPRAC compared with the FRP assay may be attributed to the fact that the CUPRAC assay measures both the actions of lipophilic and hydrophilic antioxidants, while FRP/FRAP assays can only detect hydrophilic compounds [66]. Finally, the PBP-extractable fraction showed a good ability to quench DPPH radicals (16.71 µmol/g TE), with results that are fully consistent with the extractable fraction for Turkish commercial bee-collected pollen samples (mean value 15.17 µmol/g TE) [64]. On the other hand, the bound fraction exhibited a significantly lower capability to neutralize free radicals compared with the Turkish samples.

## Figures and Tables

**Table 1:** General phytochemical composition (mean value ± st. dev.) of PBP extracts.

Assay Sample	TCC ^1^ [µg/g dw]	TPC [mg/g GAE dw]	TFC [mg/g QE dw]	HCA [mg/g CGAE dw]
I	/	11.59 ± 0.24 ^a^	12.82 ± 1.36	5.96 ± 0.04 ^a^
II	/	2.46 ± 0.07 ^b^	n.d.	1.14 ± 0.03 ^b^
III	65.05 ± 0.71	/	/	/

^1^ TCC—total carotenoid content; TPC—total phenolic content; TFC—total flavonoid content; HCA—total hydroxycinnamic-acid-derivative content; dw—dry weight; n.d.—not detected; GAE—gallic-acid equivalents; QE—quercetin equivalents; CGAE—chlorogenic-acid equivalents; I—extractable fraction; II—bound fraction; III—acetonic extract for TCC determination. Different lowercase letters in the same column denote a significant difference according to *t*-tests (*p* < 0.05).

**Table 2:** Characterization and quantification (µg/kg) of phenolic compounds in extractable and bound corn poppy pollen fractions using UHPLC-QToF-MS. Target compounds, expected retention time (t_R_), base peak, molecular formula, calculated mass, exact mass and MS^2^ fragments are presented.

t_R_	Compound Name	Formula	Calculated Mass	*m*/*z* Exact Mass	mDa	MS^2^ Fragments (% Base PEAKS)	I (µg/kg dw)	II (µg/kg dw)
Organic acid								
0.80	Citric acid ^b^	C_6_H_7_O_7_^−^	191.0197	191.0211	−1.38	103 (1), 111 (100), 112 (6)	1464.18	8.64
0.67	Gluconic acid ^b^	C_6_H_11_O_7_^−^	195.0510	195.0527	−1.69	100 (25), 101 (82), 102 (6), 102 (3), 104 (7), 105 (6), 110 (7), 111 (8), 129 (100), 130 (4), 141 (6), 141 (6), 195 (12)	1915.16	/
Phenolic acid and derivatives								
6.06	Benzoic acid ^b^	C_7_H_5_O_2_^−^	121.0295	121.0303	−0.82	/	28.70	251.96
4.25	Hidroxybenzoic acid isomer I ^b^	C_7_H_5_O_3_^−^	137.0244	137.0253	−0.85	/	38.12	98.04
8.15	Hidroxybenzoic acid isomer II ^b^	C_7_H_5_O_3_^−^	137.0244	137.0258	−1.34	/	2.69	1.37
2.49	Dihidroxybenzoic acid isomer I ^a^	C_7_H_5_O_4_^−^	153.0193	153.0213	−1.93	108 (100), 109 (84), 110 (7)	143.41	10.35
6.84	Dihidroxybenzoic acid isomer II ^a^	C_7_H_5_O_4_^−^	153.0193	153.0203	−0.95	107 (100), 108 (10), 109 (8), 111 (2), 123 (5), 125 (9), 151 (54), 153 (5)	153.77	/
7.31	Diethoxybenzoate isomer I ^b^	C_11_H_13_O_4_^−^	209.0819	209.0833	−1.35	101 (24), 103 (77), 106 (15), 106 (12), 117 (58), 118 (20), 118 (16), 119 (100), 120 (15), 121 (12), 122 (19), 129 (10), 143 (18), 150 (31)	13.05	/
9.77	Diethoxybenzoate isomer II ^b^	C_11_H_13_O_4_^−^	209.0819	209.0857	−3.74	100 (56), 116 (67), 120 (69), 120 (57), 136 (57), 141 (60), 209 (100)	/	5.81
7.15	*p*−Coumaric acid ^a^	C_9_H_7_O_3_^−^	163.0401	163.0412	−1.15	104 (2), 117 (8), 119 (100), 120 (11)	567.10	335.32
6.13	Aesculetin ^c^	C_9_H_5_O_4_^−^	177.0193	177.0212	−1.85	105 (61), 106 (10), 107 (16), 107 (10), 108 (5), 117 (7), 121 (12), 122 (4), 133 (37), 134 (63), 135 (100), 136 (8), 148 (4), 149 (17), 177 (8)	/	84.58
6.34	Caffeic acid ^a^	C_9_H_7_O_4_^−^	179.0344	179.0397	−5.30	106 (4), 107 (10), 108 (4), 109 (2), 117 (7), 133 (2), 134 (71), 135 (100), 136 (10)	7.51	/
11.85	Benzyl caffeate ^d^	C_16_H_13_O_4_^−^	269.0814	269.0900	−8.58	106 (6), 132 (1), 133 (59), 134 (100), 135 (13), 161 (19), 161 (2), 162 (2), 183 (2), 197 (7)	2.50	/
7.89	Ferulic acid ^a^	C_10_H_9_O_4_^−^	193.0506	193.0526	−1.96	106 (13), 108 (5), 108 (4), 117(8), 117(9), 118(3), 130(5), 131(3), 132 (4), 133 (57), 134 (100), 135 (8), 148 (3)	/	125.32
8.49	5−Carboxyvanillic acid ^b^	C_9_H_7_O_6_^−^	211.0248	211.0271	−2.32	107 (100), 108 (7), 108 (1), 109 (2), 123 (1), 151 (61), 152 (6)	/	610.81
Flavone								
11.80	Chrysin ^a^	C_15_H_9_O_4_^−^	253.0501	253.0541	−4.01	101 (6), 107 (25), 119 (22), 143 (54), 144 (8), 145 (21), 151 (10), 165 (8), 167 (8), 180 (6), 181 (12), 185 (7), 209 (18), 253 (100), 254 (21)	70.53	/
8.49	Tricetin ^e^	C_15_H_9_O_7_^−^	301.0354	301.0400	−4.64	109 (12), 133 (100), 134 (11), 135 (29), 137 (37), 139 (28), 165 (22), 167 (21), 175 (7), 192 (7), 201 (8), 227 (7), 255 (15), 301 (45), 302 (10)	3048.97	/
9.37	Luteolin ^e^	C_15_H_9_O_6_^−^	285.0405	285.0446	−4.11	107 (15), 121 (3), 133 (100), 134 (10), 149 (13), 151 (32), 152 (3), 175 (15), 199 (11), 201 (6), 217 (7), 241 (3), 243 (3), 285 (46), 286 (10)	4398.15	209.24
7.00	Apigenin 6,8−di−*C*−glucoside ^e^	C_27_H_29_O_15_^−^	593.1506	593.1572	−6.58	133 (100), 133 (14), 134 (14), 135 (16), 179 (25), 299 (12), 300 (12), 353 (54), 383 (35), 473 (47), 503 (13)	200.17	/
Flavanone and derivatives								
12.00	Pinocembrin ^a^	C_15_H_11_O_4_^−^	255.0663	255.0693	−3.00	107 (98), 108 (27), 135 (19), 136 (25), 145 (89), 151 (100), 169 (21), 171 (88), 172 (28), 183 (16), 185 (37), 211 (19), 213 (65), 255 (60)	345.60	/
12.23	Pinobanksin 3−*O*−acetate ^f^	C_17_H_13_O_6_^−^	313.0718	313.0751	−3.29	107 (4), 143 (6), 145 (3), 151 (2), 165 (2), 181 (2), 185 (2), 197 (5), 209 (6), 211 (2), 211 (2), 253 (100), 254 (22), 255 (3), 271 (5)	179.90	/
Flavonols and derivatives								
10.24	Kaempferol ^a^	C_15_H_9_O_6_^−^	285.0405	285.0437	−3.24	107 (8), 108 (5), 133 (10), 143 (7), 151 (8), 157 (6), 159 (8), 171 (7), 185 (12), 187 (10), 211 (8), 229 (10), 239 (8), 285 (100), 286 (23)	414.85	364.717
11.73	Kaempferol−methyl−ether ^g^	C_16_H_11_O_6_^−^	299.0561	299.0596	−3.44	107 (5), 111 (8), 119 (27), 135 (15), 143 (6), 145 (4), 151 (10), 176 (33), 178 (100), 180 (11), 185 (5), 187 (37), 188 (6), 193 (11), 297 (9)	/	<LOQ
8.43	Kaempferol 7−*O*−hexoside ^g^	C_21_H_19_O_11_^−^	447.0927	447.1009	−8.16	151 (1), 284 (5), 285 (100), 286 (20), 287 (3)	412.69	/
8.16	Kaempferol 3−*O*−hexoside ^g^	C_21_H_19_O_11_^−^	447.0927	447.1020	−9.34	151 (3), 227 (19), 228 (3), 255 (36), 256 (13), 257 (3), 284 (100), 285 (42), 286 (7), 300 (4), 301 (3), 327 (2), 447 (15), 448 (5)	232.39	/
6.53	Kaempferol 3−*O*−(6″−pentosyl)hexoside ^g^	C_26_H_27_O_15_^−^	579.1350	579.1426	−7.61	283 (3), 284 (100), 285 (23), 339 (10)	710.39	/
7.82	Kaempferol 3−*O*−(2″−pentosyl)hexoside ^g^	C_26_H_27_O_15_^−^	579.1350	579.1444	−9.42	227 (4), 255 (8), 256 (3), 284 (100), 285 (36), 429 (3)	885.46	700.168
7.99	Kaempferol 3−*O*−(6″−rhamnosyl)hexoside ^g^	C_27_H_29_O_15_^−^	593.1506	593.1576	−6.99	178 (2), 227 (3), 255 (6), 256 (2), 284 (100), 285 (30), 286 (5), 429 (3)	103.68	74.06
7.51	Kaempferol 3,7−di−*O*−hexoside ^g^	C_27_H_29_O_16_^−^	609.1456	609.1526	−6.96	255 (5), 256 (1), 283 (31), 284 (8), 285 (12), 286 (2), 446 (27), 447 (18), 448.09702(4), 489 (2), 609 (100)	714.93	311.17
8.36	Kaempferol 3−*O*−(6″−pentosyl)acetyl−hexoside ^g^	C_28_H_29_O_16_^−^	621.1456	621.1550	−9.39	151 (2), 227 (4), 255 (7), 256 (3), 284 (100), 285 (27), 286 (6), 286 (4), 435 (7)	/	24.75
8.02	Kaempferol 3−*O*−(2″−hexosyl)acetyl−hexoside ^g^	C_29_H_31_O_17_^−^	651.1561	651.1621	−5.98	227 (4), 255 (9), 256 (2), 283 (11), 284 (100), 285 (42), 286 (7), 429 (2), 471 (4), 488 (6), 489 (3), 609 (2)	1.99	21.40
7.04	Kaempferol 3−*O*−(2″−hexosyl−6″−pentosyl)hexoside ^g^	C_32_H_37_O_20_^−^	741.1878	741.1950	−7.16	116 (2), 116 (3), 151 (2), 255 (3), 283 (2), 284 (44), 285 (23), 286 (5), 561 (4), 625 (5), 741 (100)	323.07	/
7.10	Kaempferol 3−*O*−(2″,6″−di−hexosyl)hexoside ^g^	C_33_H_39_O_21_^−^	771.1984	771.2073	−8.92	179 (1), 227 (1), 255 (3), 284 (23), 285 (23), 286 (3), 429 (2), 591 (2), 609 (7), 771 (100)	36.42	/
8.28	Quercetin 3−*O*−pentoside ^g^	C_20_H_17_O_11_^−^	433.0771	433.0825	−5.37	133 (1), 165 (1), 300 (3), 301 (100), 302 (20), 303 (3)	157.22	/
7.89	Quercetin 3−*O*−hexoside ^g^	C_21_H_19_O_12_^−^	463.0877	463.0920	−4.29	151 (4), 179 (3), 243 (1), 255 (6), 256 (1), 271 (11), 272 (3), 273 (1), 300 (100), 301 (46), 302 (9), 303 (1), 463 (3)	23.07	/
7.47	Quercetin 3−*O*−(6″−pentosyl)hexoside ^g^	C_26_H_27_O_16_^−^	595.1299	595.1384	−8.53	178 (2), 255 (2), 271 (5), 299 (2), 300 (100), 301 (30)	487.11	/
7.21	Quercetin 3−*O*−(2″−hexosyl)hexoside ^g^	C_27_H_29_O_17_^−^	625.1405	625.1498	−9.29	151 (1), 178 (4), 255 (2), 271 (5), 299 (3), 300 (100), 301 (38), 302 (7), 303 (1), 445 (2), 463 (11)	577.59	/
9.22	Isorhamnetin ^h^	C_16_H_11_O_7_^−^	315.0505	315.0553	−4.83	134 (6), 136 (31), 165 (6), 199 (6), 200 (7), 201 (9), 202 (7), 216 (8), 227 (8), 228 (12), 243 (6), 272 (9), 299 (7), 300 (100), 301 (22)	321.30	/
8.29	Isorhamnetin 3−*O*−hexoside ^h^	C_22_H_21_O_12_^−^	477.1033	477.1120	−8.67	215 (1), 243 (2), 255 (4), 271 (17), 272 (6), 299 (100), 300 (48), 301 (9), 302 (1), 314 (49), 315 (21), 316 (4), 462 (4)	<LOQ	/
7.82	Isorhamnetin 3−*O*−(2″−pentosyl)hexoside ^h^	C_27_H_29_O_16_^−^	609.1456	609.1538	−8.24	209 (2), 271 (6), 272 (2), 299 (44), 300 (20), 301 (3), 313 (2), 314 (100), 315 (44), 316 (8), 429 (5)	53.85	1.78
7.69	Isorhamnetin 3−*O*−(2″−hexosyl)rhamnoside ^h^	C_28_H_31_O_16_^−^	623.1612	623.1695	−8.28	209 (2), 271 (4), 272 (2), 299 (40), 300 (19), 301 (9), 314 (100), 315 (29), 316 (5), 459 (3)	/	<LOQ
8.29	Isorhamnetin 3−*O*−(2″−rhamnosyl)hexoside ^h^	C_28_H_31_O_16_^−^	623.1612	623.1699	−8.70	137 (2), 271 (4), 299 (47), 300 (17), 301 (5), 313 (2), 314 (100), 315 (35), 316 (7), 443 (4)	/	<LOQ
7.42	Isorhamnetin 3−*O*−(2″−hexosyl)hexoside ^h^	C_28_H_31_O_17_^−^	639.1561	639.1619	−5.84	209 (2), 271 (5), 272 (1), 299 (41), 300 (19), 301 (3), 313 (2), 314 (100), 315 (43), 316 (8), 459 (5), 624 (2)	<LOQ	/
7.34	Isorhamnetin 3−*O*−(2″−hexosyl−6″−pentosyl)hexoside ^h^	C_33_H_39_O_21_^−^	771.1984	771.2048	−6.43	209 (1), 271 (2), 299 (12), 300 (7), 313 (1), 314 (24), 315 (17), 316 (3), 459 (2), 756 (1), 771 (100)	47.42	/
Other phenolics								
7.01	(Epi)catechin 3−*O*−gallate ^g^	C_22_H_17_O_10_^−^	441.0827	441.0853	−2.55	123 (28), 125 (32), 151 (16), 163 (15), 178 (35), 179 (41), 189 (62), 219 (32), 231 (32), 255 (37), 261 (17), 299 (28), 341 (16), 343 (100), 344 (22)	/	50.81
TOTAL							18,083.0	3290.3

Abbreviations: I—extractable fraction of PBP; II—bound fraction of PBP; ^a^ compounds expressed using available standards; ^b^ compounds expressed as gentisic-acid equivalents; ^c^ compounds expressed as coumaric-acid equivalents; ^d^ compounds expressed as caffeic-acid equivalents; ^e^ compounds expressed as chrysin equivalents; ^f^ compounds expressed as pinocembrin equivalents; ^g^ compounds expressed as quercetin equivalents; ^h^ compounds expressed as isorhamnetin equivalents. /—nonidentified phenolic compounds.

**Table 3:** Characterization of detected anthocyanins in extractable and bound corn poppy pollen fractions using UHPLC-QToF-MS. Target compounds, expected retention time (t_R_), base peak, molecular formula, calculated mass, exact mass and MS^2^ fragments are presented.

t_R_	Base Fragment	Formula	Calculated Mass	ppm	mDa	Compound Name	*m*/*z* Exact Mass	MS^2^ Fragments	I		II
8.53	287.0553	C_21_H_21_O_11_^+^	449.1084	6.04	2.71	Cyanidin 3-*O*-glucoside	449.1111	287 (100), 288, 289	/		+
6.57	287.0549	C_26_H_29_O_15_^+^	581.1506	4.05	2.35	Cyanidin 3-*O*-(6″-pentosyl)hexoside isomer I	581.153	287 (100), 288, 289	+		/
7.81	287.0549	C_26_H_29_O_15_^+^	581.1506	4.05	2.35	Cyanidin 3-*O*-(6″-pentosyl)hexoside isomer II	581.1531	287 (100), 288, 289	/		+
7.30	303.0503	C_27_H_31_O_17_^+^	627.1561	0.92	0.58	Delphinidin 3-*O*-(6″-*O*-hexosyl)hexoside	627.1563	303 (100), 304, 305, 145, 127	+		/

Abbreviations: I—extractable fraction of PBP; II—bound fraction of PBP.

**Table 4:** Phenylamides in extractable and bound corn poppy pollen fractions using UHPLC-QtoF-MS. Target compounds, expected retention time (t_R_), base peak, molecular formula, calculated mass, exact mass and MS^2^ fragments are presented.

t_R_	Base Fragment	Formula	Calculated Mass	ppm	mDa	Compound Name	*m*/*z* Exact Mass	MS^2^ Fragments	I	II
5.74	147.0444	C_13_H_19_N_2_O_2_^+^	235.1447	−4.48	−1.05	Coumaroyl putrescine	235.1436	147 (100), 119, 148, 120, 149	+	
4.52	100.0000	C_12_H_29_N_4_O^+^	245.2341	−3.82	−0.94	Acetyl spermine	245.2332	100 (100), 112, 113, 101, 129, 171	+	
3.24	163.0381	C_13_H_19_N_2_O_3_^+^	251.1396	2.12	0.53	Caffeoyl putrescine isomer I	251.1401	163 (100), 135, 145, 117, 164, 120, 107, 146, 136, 118, 165	+	
4.29	163.0385	C_13_H_19_N_2_O_3_^+^	251.1396	2.12	0.53	Caffeoyl putrescine isomer II	251.1401	163 (100), 135, 145, 117, 164, 146, 107, 136, 118, 165, 121	+	
1.40	147.0438	C_16_H_26_N_3_O_2_^+^	292.2025	0.68	0.2	Coumaroyl spermidine	292.2027	147 (100), 119, 204, 148, 112, 205, 120, 129, 149	+	
2.16	177.0555	C_17_H_28_N_3_O_3_^+^	322.2131	−2.38	−0.77	Feruloyl spermidine	322.2123	177 (100), 145, 234, 178, 117, 146, 149, 112, 235, 129, 146	+	
2.35	100.0764	C_21_H_35_N_4_O_3_^+^	391.2709	−1.06	−0.42	Coumaroyl acetyl spermine isomer I	391.2705	100 (100), 204, 147, 171, 129, 205, 275, 112, 148, 172, 119, 276, 155, 130, 206	+	
3.16	100.0764	C_21_H_35_N_4_O_3_^+^	391.2709	−1.06	−0.42	Coumaroyl acetyl spermine isomer II	391.2705	100 (100), 204, 147, 171, 205, 275, 129, 101, 112, 148, 172, 276, 119, 206	+	
4.51	100.0756	C_21_H_35_N_4_O_3_^+^	391.2709	−1.06	−0.42	Coumaroyl acetyl spermine isomer III	391.2705	100 (100), 204, 147, 171, 205, 275, 129, 101, 112, 148, 172, 276, 119, 206	+	
5.50	204.1032	C_21_H_35_N_4_O_3_^+^	391.2709	−1.06	−0.42	Coumaroyl acetyl spermine isomer IV	391.2705	204 (100), 147, 100, 205, 171, 129, 275, 245, 112, 148, 119, 276, 391, 172, 155	+	
8.00	147.0442	C_21_H_35_N_4_O_3_^+^	391.2709	−1.06	−0.42	Coumaroyl acetyl spermine isomer V	391.2705	147 (100), 204, 100, 171, 129, 112, 245, 275, 205, 148, 374, 119, 228, 391, 276, 246, 172, 154	+	
2.80	100.0760	C_21_H_35_N_4_O_4_^+^	407.2658	1.4	0.57	Caffeoyl acetyl spermine	407.2664	100 (100), 220, 163, 171, 291, 221, 129, 112, 101, 172, 164, 292, 145, 222, 130, 166, 212, 135, 144, 155, 113	+	
9.21	177.0538	C_23_H_27_N_2_O_5_^+^	411.192	3.66	1.5	Coumaroyl feruloyl putrescine	411.1935	177 (100), 147, 145, 178, 148, 218, 235, 117, 414, 121, 119, 370, 146, 109, 149, 119, 146, 107, 414, 265	+	
6.60	100.0768	C_23_H_37_N_4_O_4_^+^	433.2815	3.74	1.62	Coumaroyl diacetyl spermine isomer I	433.2831	100 (100), 147, 171, 204, 287, 433, 288, 172, 148, 101, 434, 129, 119, 317, 205, 270, 188, 269, 275		+
6.87	100.0771	C_23_H_37_N_4_O_4_^+^	433.2815	3.74	1.62	Coumaroyl diacetyl spermine isomer II	433.2831	100 (100), 147, 171, 204, 287, 433, 148, 288, 172, 101, 434, 205, 119, 416, 317, 120, 188, 112, 270, 275, 203		+
7.15	100.0762	C_23_H_37_N_4_O_4_^+^	433.2815	18.51	8.02	Coumaroyl diacetyl spermine isomer III	433.2895	100 (100), 147, 171, 204, 287, 416, 433, 417, 317, 270, 205, 172, 148, 288, 112, 434, 101, 373, 119, 269, 154	+	
7.82	147.0442	C_25_H_32_N_3_O_4_^+^	438.2393	4.38	1.92	Dicoumaroyl spermidine	438.2412	147 (100), 204, 292, 205, 275, 218, 148, 293, 438, 119, 221, 129, 421, 112, 276, 146, 439, 203	+	+
9.23	177.0541	C_24_H_29_N_2_O_6_^+^	441.2026	3.71	1.64	Diferuloyl putrescine	441.2042	177 (100), 145, 178, 116, 265, 117, 146, 248, 163, 444, 149, 441, 149, 443, 266, 241, 179, 136, 133	+	
7.89	204.1019	C_30_H_41_N_4_O_5_^+^	537.3077	11.55	6.2	Dicoumaroyl acetyl spermine	537.3139	204 (100), 391, 537, 147, 275, 538, 392, 205, 171, 520, 276, 539, 129, 245, 100, 317, 148, 373, 374, 112, 519, 119, 521, 393, 203	+	+
8.05	567.3178	C_31_H_43_N_4_O_6_^+^	567.3183	8.35	4.74	Coumaroyl feruloyl acetyl spermine	567.323	567 (100), 204, 568, 177, 391, 421, 234, 147, 275, 205, 392, 422, 569, 171, 145, 305, 550, 245, 235, 129, 178	+	
9.12	433.2816	C_32_H_43_N_4_O_6_^+^	579.3183	5.77	3.34	Dicoumaroyl diacetyl spermine	579.3216	433 (100), 416, 434, 147, 204, 519, 287, 417, 171, 100, 415, 313, 520, 435, 537, 317, 275, 148, 205	+	+
9.93	438.2377	C_34_H_38_N_3_O_6_^+^	584.2761	1.26	0.74	Tricoumaroyl spermidine	584.2768	438 (100), 204, 147, 439, 420, 292, 275, 421, 585, 440, 205, 218, 293, 586, 130, 148, 422, 276, 119, 318	+	
9.07	601.3018	C_35_H_43_N_4_O_5_^+^	599.3233	2.76	1.65	Dicoumaroyl benzoyl spermine	599.325	601 (100), 599, 204, 602, 453, 275, 233, 600, 147, 162, 454, 276, 205, 234, 435, 203, 379, 603, 436, 129, 148, 105	+	
8.65	641.3340	C_37_H_45_N_4_O_6_^+^	641.3339	4.82	3.09	Tricoumaroyl spermine	641.3370	641 (100), 275, 204, 642, 495, 147, 496, 276, 643, 477, 203, 205, 478, 129, 421, 497, 644, 148, 112, 119, 349	+	+
9.67	537.3072	C_39_H_47_N_4_O_7_^+^	683.3445	4.57	3.13	Tricoumaroyl acetyl spermine	683.3476	537 (100), 538, 204, 519, 391, 520, 147, 275, 392, 374, 521, 205, 276, 417, 683	+	+
9.87	369.2245	C_40_H_49_N_4_O_8_^+^	713.355	1.63	1.16	Dicoumaroyl acetyl feruloyl spermine	713.3562	369 (100), 537, 370, 567, 538, 568, 367, 519, 177, 204, 275, 391, 520, 549, 539, 550, 569, 368, 147	+	
10.42	641.3330	C_46_H_51_N_4_O_8_^+^	787.3707	3.57	2.81	Tetracoumaroyl spermine	787.3735	641 (100), 642, 623, 275, 643, 204, 624, 495, 478, 147, 322, 276, 477, 625, 479	+	
Nonidentified phenylamide										
5.94	315.1083	/	/	/	/	Coumaroyl phenylamide derivatives	330.1366	315 (100), 297, 330, 298, 147, 314, 296, 152, 316, 312, 190, 129, 331, 204, 271, 299, 123, 280, 188, 282, 269, 171, 137	+	
9.71	147.0443	/	/	/	/	Coumaroyl phenylamide derivatives	342.1786	147 (100), 119, 204, 148, 100, 112, 171, 129, 120, 205, 245, 175	+	

Abbreviations: I—extractable fraction of PBP; II—bound fraction of PBP.

**Table 5:** Isoquinoline alkaloids detected in extractable and bound corn poppy pollen fractions using UHPLC-QToF-MS. Target compounds, expected retention time (t_R_), base peak, molecular formula, calculated mass, exact mass and MS^2^ fragments are presented.

t_R_	Base Fragment	Formula	Calculated Mass	ppm	mDa	Compound Name	*m*/*z* Exact Mass	MS^2^ Fragments	I	II
Benzylisoquinoline alkaloids										
5.88	107.0498	C_16_H_18_NO_3_^+^	272.1287	−2.09	−0.57	Norcoclaurine	272.1281	107 (100), 143, 161, 108, 115, 123, 145, 209, 194, 237, 144, 191, 127, 240, 162, 133, 121, 131, 117, 181, 164, 116, 149, 226, 219, 255, 147	+	
6.78	107.0498	C_17_H_20_NO_3_^+^	286.1443	3.43	0.98	Coclaurine	286.1453	107 (100), 100, 143, 108, 209, 175, 237, 115, 137, 194, 191, 145, 160, 254, 171, 131, 144, 181, 219, 238, 210, 239, 154, 121, 176, 178	+	
6.76	107.0500	C_18_H_22_NO_3_^+^	300.16	3.44	1.03	*N*−methylcoclaurine	300.1610	107 (100), 237, 175, 143, 108, 209, 197, 137, 121, 145, 115, 269, 238, 131, 160, 254, 191, 194, 179, 163, 144, 176, 178, 239, 225	+	+
6.41	123.0440	C_18_H_22_NO_4_^+^	316.1549	8.59	2.72	3′−Hydroxy−*N*−methylcoclaurine	316.1576	123 (100), 192, 143, 175, 137, 177, 193, 253, 207, 115, 124, 161, 225, 179, 176, 178, 144, 285, 213, 160, 235, 149, 241	+	
7.13	192.1013	C_19_H_24_NO_4_^+^	330.1705	2.02	0.67	Reticuline	330.1712	192 (100), 137, 143, 178, 123, 175, 151, 206, 193, 330, 189, 177, 138, 299, 179, 180, 152, 167, 115, 176, 285, 267, 227, 239, 207, 255, 149, 145	+	+
8.30	192.1013	C_20_H_22_NO_4_^+^	340.1549	3.58	1.22	Papaverine	340.1561	192 (100), 165, 193, 340, 150, 149, 166, 177, 190, 176, 341, 292, 135, 324, 105, 119, 151, 148, 293, 133, 325	+	+
8.12	192.1020	C_20_H_24_NO_4_^+^	342.1705	9.25	3.17	3,4−Dihydropapaverine	342.1737	192 (100), 165, 193, 342, 150, 177, 190, 151, 166, 176, 343, 310, 327, 148, 137, 326, 194, 105, 294, 312, +178, 133, 131	+	+
7.41	137.0609	C_20_H_26_NO_4_^+^	344.1862	0.92	0.32	Tetrahydropapaverine	344.1865	137 (100), 206, 189, 151, 192, 174, 175, 143, 282, 158, 190, 207, 138, 298, 313, 165, 193, 191, 281, 152, 159, 176, 344, 241, 253, 177	+	+
8.15	189.0784	C_20_H_20_NO_5_^+^	354.1341	7.21	2.55	Papaveraldine	354.1367	189 (100), 188, 354, 149, 206, 275, 190, 165, 355, 247, 295, 336, 265, 135, 207, 235, 175, 293, 276, 267, 177, 195, 323, 295, 150, 178, 237, 305, 321, 107, 306, 337, 324, 311	+	+
8.07	206.1170	C_21_H_28_NO_4_^+^	358.2018	2.7	0.97	Laudanosine	358.2028	206 (100), 151, 189, 207, 165, 174, 190, 158, 296, 152, 191, 327, 281, 159, 297, 312, 192, 150, 175, 136, 177, 284, 107, 145, 193, 135	+	+
Berberine alkaloids										
8.54	176.0710	C_19_H_18_NO_4_^+^	324.1236	5.61	1.82	Stylopine	324.1254	176 (100), 149, 324, 177, 119, 325, 178, 150, 174, 135, 249, 189, 277, 120, 326, 151, 188, 219, 175, 307	+	+
7.60	178.0883	C_19_H_20_NO_4_^+^	326.1392	−1.63	−0.53	Nandinine	326.1387	178 (100), 151, 326, 179, 163, 176, 119, 149, 327, 152, 311, 135, 219, 191, 177, 277, 180, 294, 136	+	+
7.45	178.0867	C_19_H_22_NO_4_^+^	328.1549	4.32	1.42	Scoulerine	328.1563	178 (100), 151, 179, 328, 163, 119, 180, 176, 329, 152, 313, 296, 137, 164, 191, 312, 177, 190, 136, 298, 135, 279	+	+
7.73	192.1018	C_20_H_22_NO_4_^+^	340.1549	4.46	1.52	Canadine	340.1564	192 (100), 193, 340, 177, 341, 190, 194, 178, 149, 191	+	
8.40	320.0908	C_20_H_20_NO_6_^+^	370.1291	3.88	1.44	Papaverrubin E	370.1305	320 (100), 321, 338, 177, 352, 176, 322, 292, 353, 174, 339, 149, 190, 303, 293, 291, 262, 290, 263, 135, 310, 178, 308	+	+
Other isoquinoline alkaloids										
6.50	123.0447	C_18_H_20_NO_4_^+^	314.1392	2.76	0.87	Laurolitsine	314.1401	123 (100), 298, 192, 299, 143, 175, 178, 314, 137, 151, 300, 253, 285, 179, 177, 107, 207, 193, 115, 176, 161, 315, 152, 225, 124, 213, 270, 284, 316, 235, 254, 283, 241, 227, 255, 237, 256, 209, 301, 286, 282, 296, 223, 252	+	
8.74	352.1181	C_21_H_22_NO_6_^+^	384.1447	3.61	1.39	Hydrastine	384.1461	352 (100), 190, 320, 353, 188, 334, 303, 291, 263, 321, 189, 191, 293, 235, 176, 322, 304, 149, 335, 294, 292, 233, 324	+	+

Abbreviations: I—extractable fraction of PBP; II—bound fraction of PBP.

**Table 6:** Fatty-acid composition (%) obtained from GC-FID analysis of lipid PBP fraction.

Fatty Acid (FA)	% of Total Fatty Acids
Capric acid (C10:0)	0.91 ± 0.14 ^h^
Palmitic acid (C16:0)	10.92 ± 0.43 ^d^
Stearic acid (C18:0)	13.72 ± 0.78 ^c^
Oleic acid (C18:1)	10.73 ± 0.74 ^d^
Linoleic acid (C18:2)	7.25 ± 0.48 ^e^
α-Linolenic acid (C18:3)	22.98 ± 0.82 ^b^
Eicosadienoic acid (C20:2)	5.20 ± 0.22 ^f^
Erucic acid (C22:1)	3.01 ± 0.12 ^g^
Docosahexaenoic acid (C22:3)	25.26 ± 0.62 ^a^
Total SFAs *	25.55
Total UFAs	74.45

* SFAs—saturated fatty acids; UFAs—unsaturated fatty acids. Different lowercase letters in the column indicate a significant difference according to *t*-test (*p* < 0.05).

**Table 7:** Antioxidant properties (mean value ± st. dev.) of PBP extracts.

Assay Sample	TAC ^1^ [mg/g AAE dw]	FRP [mg/g AAE dw]	CUPRAC [mg/g AAE dw]	DPPH^∙^ [µmol/g TE dw]
I	28.92 ± 1.06 ^a^	5.58 ± 0.04 ^a^	69.00 ± 0.96 ^a^	16.71 ± 0.87 ^a^
II	0.92 ± 0.01 ^b^	0.35 ± 0.03 ^b^	22.78 ± 0.66 ^b^	2.94 ± 0.12 ^b^

^1^ TAC—in vitro phosphomollybdenum total antioxidant capacity; FRP—Ferric-Reducing Power; CUPRAC—Cupric-Reducing Antioxidant Capacity; DPPH^∙^—2,2-diphenyl-1-picrylhydrazyl radical; dw—dry weight; AAE—ascorbic-acid equivalents; TE—Trolox equivalents; I—extractable fraction; II—bound fraction. Different lowercase letters in the same column denote a significant difference among samples according to *t*-test (*p* < 0.05).

## Data Availability

Data available on request from the authors.

## Supplementary Materials

The following supporting information can be downloaded at: https://www.mdpi.com/article/10.3390/antiox12071424/s1, Table S1: Equation parameters and correlation coefficient (R^2^) used for phenolic standards.

## References

[1] Temple N.J. (2022). A Rational Definition for Functional Foods: A Perspective. Front. Nutr..

[2] Li Q.-Q., Wang K., Marcucci M.C., Sawaya A.C.H.F., Hu L., Xue X.-F., Wu L.-M., Hu F.-L. (2018). Nutrient-Rich Bee Pollen: A Treasure Trove of Active Natural Metabolites. J. Funct. Foods.

[3] Thakur M., Nanda V. (2020). Composition and Functionality of Bee Pollen: A Review. Trends Food Sci. Technol..

[4] Kostić A.Ž., Milinčić D.D., Barać M.B., Ali Shariati M., Tešić Ž.L., Pešić M.B. (2020). The Application of Pollen as a Functional Food and Feed Ingredient—The Present and Perspectives. Biomolecules.

[5] Ulusoy E., Kolayli S. (2014). Phenolic Composition and Antioxidant Properties of Anzer Bee Pollen. J. Food Biochem..

[6] Hemmami H., ben Seghir B., ben Ali M., Rebiai A., Zeghoud S., Brahmia F. (2020). Phenolic Profile and Antioxidant Activity of Bee Pollen Extracts from Different Regions of Algeria. Ovidius Univ. Ann. Chem..

[7] Waś E., Szczęsna T., Rybak-Chmielewska H., Teper D., Jaśkiewicz K. (2017). Application of HPLC-DAD Technique for Determination of Phenolic Compounds in Bee Pollen Loads. J. Apic. Sci..

[8] Kao Y.T., Lu M.J., Chen C. (2011). Preliminary Analyses of Phenolic Compounds and Antioxidant Activities in Tea Pollen Extracts. J. Food Drug Anal..

[9] Alimoglu G., Guzelmeric E., Yuksel P.I., Celik C., Deniz I., Yesilada E. (2021). Monofloral and Polyfloral Bee Pollens: Comparative Evaluation of Their Phenolics and Bioactivity Profiles. LWT.

[10] Kostić A.Ž., Milinčić D.D., Gašić U.M., Nedić N., Stanojević S.P., Tešić Ž.L., Pešić M.B. (2019). Polyphenolic Profile and Antioxidant Properties of Bee-Collected Pollen from Sunflower (*Helianthus annuus* L.) Plant. LWT.

[11] Alcázar R., Bueno M., Tiburcio A.F. (2020). Cells Polyamines: Small Amines with Large Effects on Plant Abiotic Stress Tolerance. Cells.

[12] Chen D., Shao Q., Yin L., Younis A., Zheng B. (2019). Polyamine Function in Plants: Metabolism, Regulation on Development, and Roles in Abiotic Stress Responses. Front. Plant Sci..

[13] Ni Y.-Q., Liu Y.-S. (2021). New Insights into the Roles and Mechanisms of Spermidine in Aging and Age-Related Diseases. Aging Dis..

[14] Pietrocola F., Castoldi F., Kepp O., Carmona-Gutierrez D., Madeo F., Kroemer G. (2019). Spermidine Reduces Cancer-Related Mortality in Humans. Autophagy.

[15] Kim S.B., Liu Q., Ahn J.H., Jo Y.H., Turk A., Hong I.P., Han S.M., Hwang B.Y., Lee M.K. (2018). Polyamine Derivatives from the Bee Pollen of *Quercus mongolica* with Tyrosinase Inhibitory Activity. Bioorg. Chem..

[16] Kyselka J., Bleha R., Dragoun M., Bialasová K., Horáčková Š., Schätz M., Sluková M., Filip V., Synytsya A. (2018). Antifungal Polyamides of Hydroxycinnamic Acids from Sunflower Bee Pollen. J. Agric. Food Chem..

[17] Gardana C., Del Bo’ C., Quicazán M.C., Corrrea A.R., Simonetti P. (2018). Nutrients, Phytochemicals and Botanical Origin of Commercial Bee Pollen from Different Geographical Areas. J. Food Compos. Anal..

[18] El Ghouizi A., El Menyiy N., Falcão S.I., Vilas-Boas M., Lyoussi B. (2020). Chemical Composition, Antioxidant Activity, and Diuretic Effect of Moroccan Fresh Bee Pollen in Rats. Vet. World.

[19] Aylanc V., Tomás A., Russo-Almeida P., Falcão S.I., Vilas-Boas M. (2021). Assessment of Bioactive Compounds under Simulated Gastrointestinal Digestion of Bee Pollen and Bee Bread: Bioaccessibility and Antioxidant Activity. Antioxidants.

[20] Khongkarat P., Ramadhan R., Phuwapraisirisan P., Chanchao C. (2020). Safflospermidines from the Bee Pollen of *Helianthus annuus* L. Exhibit a Higher in Vitro Antityrosinase Activity than Kojic Acid. Heliyon.

[21] Pernal S., Currie R. (2001). The Influence of Pollen Quality on Foraging Behavior in Honeybees (*Apis mellifera* L.). Behav. Ecol. Sociobiol..

[22] Muth F., Francis J.S., Leonard A.S. (2016). Bees Use the Taste of Pollen to Determine Which Flowers to Visit. Biol. Lett..

[23] Fatrcová-Šramková K., Nôžková J., Kačániová M., Máriássyová M., Rovná K., Stričík M. (2013). Antioxidant and Antimicrobial Properties of Monofloral Bee Pollen. J. Environ. Sci. Health Part B.

[24] Zhou J., Qi Y., Ritho J., Zhang Y., Zheng X., Wu L., Li Y., Sun L. (2015). Flavonoid Glycosides as Floral Origin Markers to Discriminate of Unifloral Bee Pollen by LC–MS/MS. Food Control.

[25] Sarabandi K., Akbarbaglu Z., Peighambardoust S.H., Ayaseh A., Jafari S.M. (2023). Physicochemical, Antibacterial and Bio-Functional Properties of Persian Poppy-Pollen (*Papaver bracteatum*) Protein and Peptides. J. Food Measur. Character..

[26] Kostić A.Ž., Milinčić D.D., Nedić N., Gašić U.M., Špirović Trifunović B., Vojt D., Tešić Ž.L., Pešić M.B. (2021). Phytochemical Profile and Antioxidant Properties of Bee-Collected Artichoke (*Cynara scolymus*) Pollen. Antioxidants.

[27] Kostić A., Mačukanović-Jocić M.P., Špirović Trifunović B.D., Vukašinović I., Pavlović V.B., Pešić M.B. (2017). Fatty Acids of Maize Pollen—Quantification, Nutritional and Morphological Evaluation. J. Cereal. Sci..

[28] Shahidi F., Yeo J. (2016). Insoluble-Bound Phenolics in Food. Molecules.

[29] Shahidi F., Hossain A. (2023). Importance of Insoluble-Bound Phenolics to the Antioxidant Potential Is Dictated by Source Material. Antioxidants.

[30] Kostić A.Ž., Milinčić D.D., Stanisavljević N.S., Gašić U.M., Lević S., Kojić M.O., Tešić Ž.L., Nedović V., Barać M.B., Pešić M.B. (2021). Polyphenol Bioaccessibility and Antioxidant Properties of in Vitro Digested Spray-Dried Thermally-Treated Skimmed Goat Milk Enriched with Pollen. Food Chem..

[31] de Melo B.K.C., da Silva J.A., da Silva Gomes R.D., Custódio P.P., de Lira G.A., Ramalho A.M.Z., Gonçalves M.C., da Fonseca S.B., do Nascimento Rangel A.H., de Fátima Bezerra M. (2023). Physicochemical Composition and Functional Properties of Bee Pollen Produced in Different Locations. Braz. J. Food Technol..

[32] De-Melo A.A.M., Estevinho L.M., Moreira M.M., Delerue-Matos C., de Freitas A.d.S., Barth O.M., de Almeida-Muradian L.B. (2018). Multivariate Approach Based on Physicochemical Parameters and Biological Potential for the Botanical and Geographical Discrimination of Brazilian Bee Pollen. Food Biosci..

[33] Asmae E.G., Nawal E.M., Bakour M., Lyoussi B. (2021). Moroccan Monofloral Bee Pollen: Botanical Origin, Physicochemical Characterization, and Antioxidant Activities. J. Food Qual..

[34] Kaèániová M., Nô¦ková J., Fatrcová-Šramková K., Kropková Z., Kubincová J. (2010). Antioxidant, Antimicrobial Activity and Heavy Metals Content in Pollen of *Papaver somniferum* L.. Ecol. Chem. Eng. A.

[35] Campos M.G., Webby R.F., Markham K.R. (2002). The Unique Occurrence of the Flavone Aglycone Tricetin in Myrtaceae Pollen. Z. Naturforsh. C.

[36] Wollenweber E., Dörr M. (2008). Occurrence and Distribution of the Flavone Tricetin and Its Methyl Derivatives as Free Aglycones. Nat. Prod. Commun..

[37] Jiang T., Guo K., Liu L., Tian W., Xie X., Wen S., Wen C. (2020). Integrated Transcriptomic and Metabolomic Data Reveal the Flavonoid Biosynthesis Metabolic Pathway in *Perilla frutescens* (L.) Leaves. Sci. Rep..

[38] Liu Y., Fernie A.R., Tohge T. (2022). Diversification of Chemical Structures of Methoxylated Flavonoids and Genes Encoding Flavonoid-*O*-Methyltransferases. Plants.

[39] Negri G., Teixeira E.W., Florêncio Alves M.L.T.M., de Camargo Carmello Moreti A.C., Otsuk I.P., Borguini R.G., Salatino A. (2011). Hydroxycinnamic Acid Amide Derivatives, Phenolic Compounds and Antioxidant Activities of Extracts of Pollen Samples from Southeast Brazil. J. Agric. Food Chem..

[40] Negri G., Barreto L.M.R.C., Sper F.L., de Carvalho C., das Graças Ribeiro Campos M. (2018). Phytochemical Analysis and Botanical Origin of Apis Mellifera Bee Pollen from the Municipality of Canavieiras, Bahia State, Brazil. Braz. J. Food Technol..

[41] Aylanc V., Larbi S., Calhelha R., Barros L., Rezouga F., Rodríguez-Flores M.S., Seijo M.C., El Ghouizi A., Lyoussi B., Falcão S.I. (2023). Evaluation of Antioxidant and Anticancer Activity of Mono- and Polyfloral Moroccan Bee Pollen by Characterizing Phenolic and Volatile Compounds. Molecules.

[42] Rodríguez-Flores M.S., Escuredo O., Seijo M.C., Rojo S., Vilas-Boas M., Falcão S.I. (2023). Phenolic Profile of Castanea Bee Pollen from the Northwest of the Iberian Peninsula. Separations.

[43] Gabriele M., Parri E., Felicioli A., Sagona S., Pozzo L., Biondi C., Domenici V., Pucci L. (2015). Phytochemical Composition and Antioxidant Activity of Tuscan Bee Pollen of Different Botanic Origins. Ital. J. Food Sci..

[44] Chelucci E., Chiellini C., Cavallero A., Gabriele M. (2023). Bio-Functional Activities of Tuscan Bee Pollen. Antioxidants.

[45] Zhang X., Yu M., Zhu X., Liu R., Lu Q. (2022). Metabolomics Reveals That Phenolamides Are the Main Chemical Components Contributing to the Anti-Tyrosinase Activity of Bee Pollen. Food Chem..

[46] Lopez X., Mujika J.I., Blackburn G.M., Karplus M. (2003). Alkaline Hydrolysis of Amide Bonds: Effect of Bond Twist and Nitrogen Pyramidalization. J. Phys. Chem. A.

[47] Butnariu M., Quispe C., Herrera-Bravo J., Pentea M., Sarac I., Küşümler A.S., Özçelik B., Painuli S., Semwal P., Imran M. (2022). Papaver Plants: Current Insights on Phytochemical and Nutritional Composition Along with Biotechnological Applications. Oxid. Med. Cell Longev..

[48] Végh R., Csóka M., Sörös C., Sipos L. (2021). Food Safety Hazards of Bee Pollen—A Review. Trends Food Sci. Technol..

[49] Faisal S., Badshah S.L., Kubra B., Emwas A.-H., Jaremko M. (2023). Alkaloids as Potential Antivirals. A Comprehensive Review. Nat. Prod. Bioprospect..

[50] Yun D., Yoon S.Y., Park S.J., Park Y.J. (2021). The Anticancer Effect of Natural Plant Alkaloid Isoquinolines. Int. J. Mol. Sci..

[51] Ashrafi S., Alam S., Sultana A., Raj A., Emon N.U., Richi F.T., Sharmin T., Moon M., Park M.N., Kim B. (2023). Papaverine: A Miraculous Alkaloid from Opium and Its Multimedicinal Application. Molecules.

[52] Manani R., Kazemzadeh G., Saberi A., Sadeghipour F., Rahmani A. (2019). Effect of Local Papaverine on Arteriovenous Fistula Maturation in Patients with End-Stage Renal Disease. Braz. J. Nephrol..

[53] Kostić A.Ž., Pešić M.B., Trbović D., Petronijević R., Dramićanin A.M., Milojković-Opsenica D.M., Tešić Ž.L. (2017). The Fatty Acid Profile of Serbian Bee-Collected Pollen—A Chemotaxonomic and Nutritional Approach. J. Apic. Res..

[54] Mărgăoan R., Mărghitaş L.A., Dezmirean D.S., Dulf F.V., Bunea A., Socaci S.A., Bobiş O. (2014). Predominant and Secondary Pollen Botanical Origins Influence the Carotenoid and Fatty Acid Profile in Fresh Honeybee-Collected Pollen. J. Agric. Food Chem..

[55] Mărgăoan R., Özkök A., Keskin Ş., Mayda N., Urcan A.C., Cornea-Cipcigan M. (2021). Bee Collected Pollen as a Value-Added Product Rich in Bioactive Compounds and Unsaturated Fatty Acids: A Comparative Study from Turkey and Romania. LWT.

[56] Satranský M., Fraňková A., Kuchtová P., Pazderů K., Capouchová I. (2021). Oil Content and Fatty Acid Profile of Selected Poppy (*Papaver somniferum* L.) Landraces and Modern Cultivars. Plant Soil Environ..

[57] Melo D., Álvarez-Ortí M., Nunes M.A., Espírito Santo L., Machado S., Pardo J.E., Oliveira M.B.P.P. (2022). Nutritional and Chemical Characterization of Poppy Seeds, Cold-Pressed Oil, and Cake: Poppy Cake as a High-Fibre and High-Protein Ingredient for Novel Food Production. Foods.

[58] Senila L., Neag E., Cadar O., Kovacs M.H., Becze A., Senila M. (2020). Chemical, Nutritional and Antioxidant Characteristics of Different Food Seeds. Appl. Sci..

[59] Gavrilova V., Shelenga T., Porokhovinova E., Dubovskaya A., Kon’kova N., Grigoryev S., Podolnaya L., Konarev A., Yakusheva T., Kishlyan N. (2020). The Diversity of Fatty Acid Composition in Traditional and Rare Oil Crops Cultivated in Russia. Biol. Commun..

[60] Grauso L., de Falco B., Motti R., Lanzotti V. (2021). Corn Poppy, Papaver Rhoeas L.: A Critical Review of Its Botany, Phytochemistry and Pharmacology. Phytochem. Rev..

[61] Simopoulos A.P. (2016). An Increase in the Omega-6/Omega-3 Fatty Acid Ratio Increases the Risk for Obesity. Nutrients.

[62] Li J., Pora B.L.R., Dong K., Hasjim J. (2021). Health Benefits of Docosahexaenoic Acid and Its Bioavailability: A Review. Food Sci. Nutr..

[63] Gercek Y.C., Celik S., Bayram S. (2021). Screening of Plant Pollen Sources, Polyphenolic Compounds, Fatty Acids and Antioxidant/Antimicrobial Activity from Bee Pollen. Molecules.

[64] Dulger Altiner D., Sandikci Altunatmaz S., Sabuncu M., Aksu F., Sahan Y. (2021). *In-Vitro* Bioaccessibility of Antioxidant Properties of Bee Pollen in Turkey. Food Sci. Technol.-Camp..

[65] Bakour M., Laaroussi H., Ousaaid D., Oumokhtar B., Lyoussi B. (2021). Antioxidant and Antibacterial Effects of Pollen Extracts on Human Multidrug-Resistant Pathogenic Bacteria. J. Food Qual..

[66] Apak R., Güçlü K., Demirata B., Özyürek M., Çelik S.E., Bektaşoğlu B., Berker K.I., Özyurt D. (2007). Comparative Evaluation of Various Total Antioxidant Capacity Assays Applied to Phenolic Compounds with the CUPRAC Assay. Molecules.

