# Supplementary material for: Monofloral Corn Poppy Bee-Collected Pollen—A Detailed Insight into Its Phytochemical Composition and Antioxidant Properties
Source: Antioxidants (Basel). 2023 Jul 14;12(7):1424. doi: 10.3390/antiox12071424 (PMC10376007; doi:10.3390/antiox12071424)
Supplement: Supplementary file 1 [file antioxidants-12-01424-s001.zip › antioxidants-2495018-supplementary.pdf]

Table S1. Equation parameters and correlation coefficient (R^2) used for phenolic standards.

Standards	Y=a*X±b	R^2
Gentisic acid	Y= 1726721*X+466061.0629	0.9924
Coumaric acid	Y= 404285.2881*X+13101.3113	0.9981
Caffeic acid	Y= 3049527.6728*X+856403.3939	0.9921
Ferulic acid	Y= 172046.6074*X-47682.2769	0.9906
Chrysin	Y= 421642.8753*X+255584.8437	0.9914
Pinocembrin	Y= 156751.2871*X+40416.1819	0.997
Quercetin	Y= 769214.5670 * x + 825343.6827	0.9915
Isorhamnetin	Y= 1084944.2566 * x + 1008067.9239	0.9936
